# Connected to the spirit of the frog: An Internet-based survey on Kambô, the secretion of the Amazonian Giant Maki Frog (*Phyllomedusa bicolor*): Motivations for use, settings and subjective experiences

**DOI:** 10.1177/0269881121991554

**Published:** 2021-03-04

**Authors:** Tomislav Majić, Meike Sauter, Felix Bermpohl, Timo T Schmidt

**Affiliations:** 1 Department of Psychiatry and Psychotherapy, Charité Universitätsmedizin Berlin, Berlin, Germany; 2 Department of Psychosomatic Medicine and Psychotherapy, Helios Clinic Emil von Behring, Berlin, Germany; 3 Department of Education and Psychology, Freie Universität Berlin, Berlin, Germany

**Keywords:** Kambô, Giant Maki Frog, Sapo, Amazonas, *Phyllomedusa bicolor*, alternative medicine, motivation, Rapé, psychedelic, ayahuasca, Sananga

## Abstract

**Background/aim:**

Kambô is a name for the secretion of the Giant Maki Frog (*Phyllomedusa bicolor*), which has been used by indigenous cultures from the Amazonas basin and has recently become popular in alternative healing circles in Western countries, with a certain overlap with psychedelic self-exploration.

**Methods:**

We carried out an online-based survey in English (54.92%) and German investigating motivations for using Kambô, settings in which rituals take place, and subjective experiences during and after the application.

**Results:**

Participants (*n* = 386, mean age: 38.08 years, (standard deviation = 9.95)) were well-educated individuals with an increased lifetime prevalence of the use of ayahuasca (67.88%). A plethora of motivations for using Kambô was reported, including general healing, detoxification and spiritual growth. Acute effects included severe physical reactions and mild psychoactive effects, most surprisingly, the feeling of being connected to the frog’s spirit (41.97%), whereas predominantly positive persisting psychological effects were reported. Few participants reported long-lasting physical (2.85%) or mental (1.81%) health problems which they attributed to Kambô. Of the participants, 87.31% reported an increase in personal well-being or life satisfaction, and 64.26% considered Kambô to have been at least of ‘very much’ spiritual significance for their lives.

**Conclusions:**

The majority of users claimed beneficial effects including more health-orientated behaviors, whereas only very few participants complained about new health problems which they ascribed to Kambô. In retrospect, Kambô was given a high personal and spiritual significance by many participants. Additional research is needed to determine in how far reported effects are modulated by setting and subjective expectations.

## Introduction

During the last decade, the use of Kambô, a secretion stemming from the Amazonian Giant Maki Frog (*Phyllomedusa bicolor*), has gained increasing interest among certain complementary healing circles in the Western countries (Hesselink, 2018b). Originally, Kambô is used as an invigorating stimulant by various indigenous hunting groups from the Southwestern Amazon basin at the border between Brazil and Peru, including the Katukina, Marubo, Mayoruna and the Matsés, among others (Daly et al., 1992). In these traditions, Kambô is most commonly used as a remedy to cure hunters from ‘panema’, a term which originally refers to bad luck in hunting (De Lima and Labate, 2008). However, various other negative conditions have been subsumed to ‘panema’, making Kambô a remedy against a variety of mental and physical sufferings in both a medical and magical sense (De Lima and Labate, 2014). First ethnographical observations on Kambô have been published by the French missionary Constantin Tastevin in 1925 (Tastevin, 1925), followed by anthropological studies in the 1960s (Carneiro, 1970; De Lima and Labate, 2008). While initial ethnological (Carneiro, 1970) and basic pharmacological (Anastasi and Erspamer, 1970) research on the biochemical components of the *Phyllomedusa* skin secretion have been conducted already in the late 1960s, to date little is known about how Kambô is used today in urban centres in the Western world (Hesselink, 2018b), where it has become popular since the beginning of this century (Labate and De Lima, 2015). This is also true for the motivations of use and perceived subjective effects of Kambô by Western users.

**Figure 1.**
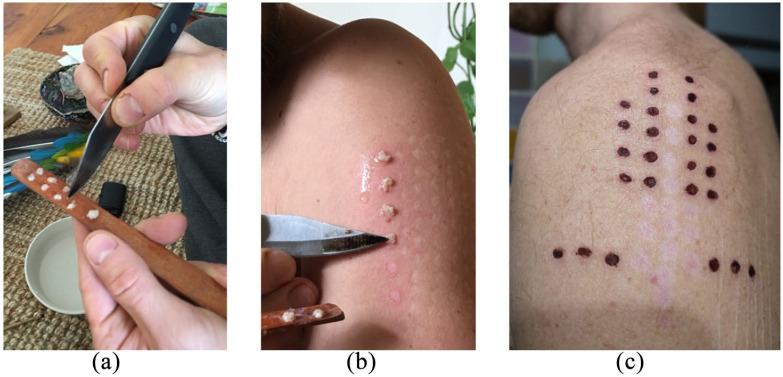
Process of preparing the secretion and application on points: (a) preparation of Kambô by a practitioner. Secretion is mixed with some water and separated in several dosages on a wooden stick, (b) the secretion is applied onto fresh superficial skin burns on the shoulder of the recipient to facilitate absorption via the lymphatic system, and (c) wounds approximately 3 days after the application: symmetric formation of 26 fresh crusts and 15 additional wounds, done on different days on the shoulder of the recipient.

In the traditional way of usage, the secretion is obtained by carefully tying the frog up and rubbing its skin with a sharp instrument, collecting the secretion on a wooden stick (Daly et al., 1992). Notably, the frog is usually not harmed, but treated respectfully, and released to its natural habitat after the procedure is finished (Den Brave et al., 2014). Probably due to its low bioavailability, the secretion is most commonly applied parenterally on a line of fresh superficial burns (‘points’) on the arms, legs or chest by the applicator (Hesselink, 2018a), leaving characteristic scars which are depicted in Figure 1. It has been reported that within minutes, a dose-dependent, often strong reaction (‘violent illness’) is induced (Daly et al., 1992), including tachycardia, sweating and heavy vomiting, which usually subsides within 60 min, followed by a state of listlessness or sleep, lasting from one day to several days (Daly et al., 1992; Hesselink and Winkelman, 2019). Subsequently, a state of increased stamina and clarity of thoughts is reported, associated with a heightened capacity for hunting (Daly et al., 1992). Due to the distinct roles of applicators and recipients (Labate and De Lima, 2015), the term ‘user’ appears to be somewhat incorrect, as self-administration is highly uncommon when compared to other substances.

Gradually, the use of Kambô has spread from the Amazonian rain forest to urban centres in Brazil, following its introduction by the rubber tapper Francisco Gomes, whose interpretation and re-invention of the Kambô practice has become one major point of reference for many urban Kambô practitioners (De Lima and Labate, 2008). Interestingly, it has been reported that the spread of Kambô from the Amazon to the urban regions has been promoted by Gomes and other members of the Brazilian ayahuasca religions like Santo Daime and União do Vegetal (UDV) (Labate and De Lima, 2015). Some of the indigenous cultures using Kambô also appear to use ayahuasca (Hesselink, 2018b), but we are not aware of any ritual associations between both practices. In contrast to ayahuasca, Kambô can be applied by non-shamans and appears to have other purposes (Hesselink and Winkelman, 2019), although potential synergies of the two techniques have been anecdotally suggested (Gorman, 2015). Since the beginning of this century, Kambô has found its way to other Western countries, particularly those in North America and Europe (Hesselink, 2018a). In the urban, non-indigenous context, Kambô has been adapted by new-age health practitioners, neo-shamans and other healers, again, with some of them being linked to the use of ayahuasca (De Lima and Labate, 2008). In this process, the Kambô ritual has undergone various changes, based on different systems of reference and users’ needs, for which the term ‘shamanization’ and ‘therapeutization’ of Kambô have been proposed (De Lima and Labate, 2014), reaching far beyond the original methods and aims of use as carried out by indigenous cultures.

Although some authors have investigated anthropological and pharmacological aspects of Kambô, to date, knowledge on motives for use, ritual frameworks and psychological effects of Kambô in Western users is scarce and remains anecdotal (De Lima and Labate, 2008). Notably, it has been controversial whether Kambô displays psychoactive properties at all. Intriguingly, strong beneficial effects on mental and physical health have anecdotally been claimed by Kambô practitioners and recipients, including beneficial effects across a broad spectrum of pathologies including, among many others, addiction, depression, Hashimoto thyroiditis, coeliac disease (Hesselink, 2018b), post-traumatic stress disorder, diabetes, infectious diseases, cancer and hypertension (Hesselink, 2018c). Of note, the peptides which have been identified in the *Phyllomedusa* secretion include substances with antibacterial and anti-protozoan properties (Leite et al., 2005), and substances with a high affinity to µ-opioid receptors, exhibiting strong analgesic properties (Hesselink and Schatman, 2018; Negri et al., 2006). Up to 16 bioactive peptides (e.g. adenoregulin, bradykinin, ceruletide, deltorphin, dermorphin, phyllomedusin, phyllokinin, sauvagine and others) have been identified in the *Phyllomedusa* secretion (De Morais et al., 2018). Among these, it has been hypothesised that ceruletide and sauvagine might be responsible for some of the central effects, whereas ceruletide, phyllokinin, phyllomedusin, sauvagine and opioid peptides might mediate peripheral effects (Erspamer et al., 1993).

Given its increasing popularity in Western urban centres, it is desirable to learn more about Kambô applicators and recipients. Therefore, we carried out an Internet-based survey investigating different aspects of use. The survey was distributed with support from Kambô practitioners, specific Kambô-related social media websites, scientists and other individuals related to the field of Kambô or ritual plants. In this survey, we addressed the following questions: (a) is Kambô usage associated with any specific participant characteristics?; (b) what are the motivations for using Kambô?; (c) how do users prepare for Kambô sessions and in which settings do rituals take place?; (d) which acute subjective effects are experienced when receiving Kambô, and how are these effects evaluated by the users?; (e) are these effects associated with dosage?; (f) which subacute and persistent effects on health are reported by users?

## Methods

### Design of online survey

The survey was hosted on SoSci Survey (https://www.soscisurvey.de/), a German online survey software tool. The survey was designed to take approximately 30 min to be completed. Survey questions were testing for participants’ demographics and basic information on the use of Kambô, ritual plants, other substances and spiritual practice; motivation, expectations and subjectively experienced benefits; acute effects of an exemplary Kambô session; and subjectively experienced long-term effects. Participants had the choice between a German and English version of the survey. A full report of the English survey items and questions can be found in Supplementary Material S1.

Formulation of items was discussed and reviewed by Kambô practitioners to match concepts and wording among German and English-speaking practitioners (e.g. ‘received Kambô’ instead of ‘Kambô administration’; ‘Kambô experience/effects’ instead of ‘acute symptoms induced by Kambô’; ‘Kambô treatment’ instead of ‘Kambô session’).

After providing basic demographic information, participants reported their age of having had received Kambô for the first time, how often and in which setting they received Kambô and when the last time had been. Participants also provided information on their use of ‘ritual plants’ (e.g. ayahuasca, psilocybin) and other substances (e.g. alcohol, cannabis, amphetamine) and if Kambô affected their usage. Further, participants provided information on practicing spiritual practices (e.g. yoga, meditation, prayers) and what importance those had for them.

In the next section of the survey, we requested information on the personal motivation for undergoing a Kambô treatment, including what the most important reason for receiving Kambô for the first time was. Participants were asked to indicate how strongly they were convinced that Kambô would have positive effects before and after they first received Kambô, if their expectations were met after the first treatment and in what ways they had potentially benefitted from it.

Next, participants were requested to report about their subjective experiences during and after one exemplary Kambô session, where this exemplary session was defined as one that they remembered best or that was especially meaningful. Information on the Kambô dosage (number of ‘dots’), the set and setting, duration of the experience, acute effects and long-term effects were requested as well as information on the potential usage of ritual plants or recreational drugs preceding the session. The acute subjective experiences during the Kambô session were assessed using items of the well-established and validated Mystical Experience Questionnaire (MEQ) (MacLean et al. 2012) and the Challenging Experience Questionnaire (CEQ) (Barrett et al., 2016).

In the last section, participants were asked to focus on the overall effects that Kambô has had on their life. They were asked questions about positive long-term effects of Kambô (e.g. improvement of mental clarity, spiritual growth or overall wellbeing), effects on their physical and mental health (e.g. improvement, deterioration) and on their intake of foodstuffs and substances (e.g. medication, recreational drugs). They were also asked if they ever regretted participating in a Kambô treatment and to what extent the following statement applies ‘My life has been influenced in a lasting and profound way by Kambô’. The survey also contained selected questions from the Persisting Experience Questionnaire (PEQ) (Griffiths et al., 2006), e.g. (a) How personally meaningful was the experience? (b) Indicate the degree to which the experience was spiritually significant to you? (c) Do you believe that the experience and your contemplation of that experience have led to a change in your current sense of personal well-being or life satisfaction?

### Recruitment of participants

Participants were recruited primarily via email invitation distributed by national and international Kambô experts and by Internet advertisement on social media networking sites (Facebook), websites on psychoactive substances (e.g. www.asdb.info) and social news aggregation (Reddit). The posted Internet link led participants to a website, giving basic information on the study and leading to the survey. Advertisement cards were provided at healing festivals in the surrounding areas of Berlin, Germany.

The study was advertised as a survey on the subjective experiences of Kambô. Potential volunteers were informed that participating was anonymous and that skipping questions was possible, and they were asked to answer the questions as honestly and as precisely as possible.

### Inclusion/exclusion criteria

Only datasets were included where participants reported that they actually had a Kambô treatment and they were at least 18 years old at the time of the treatment. Please note that these criteria were also made explicit in the recruitment texts for the study. Further, datasets with less than 75% of all items being answered and where the overall time for filling out the questionnaire was less than 10 min were excluded from the analysis.

### Analyses

For those items where open responses were allowed in addition to responses on pre-defined categories, where possible we integrated the responses in the provided pre-defined categories. Additionally we classified the remaining responses into new categories, which are reported, wherever a specific motive was named at least two times.

To test for dose-response relationships we calculated Spearman correlation coefficients for the effects of the ‘number of dots’ on the measures of ‘duration of effects’ and the included MEQ and CEQ items.

## Results

### Sample characteristics and associations with Kambô usage

We obtained *n* = 439 datasets, where *n* = 386 were included in the analysis after application of the exclusion criteria (English: *n* = 212, 54.92%; German *n* = 174, 45.08%). Of the English survey participants *n* = 56 reported to be from the USA, *n* = 27 UK, *n* = 19 Germany, *n* = 14 Australia, as the most commonly named nationalities. Further sample characteristics are listed in Table 1.

**Table 1.** Sample characteristics. Characteristics of the analysed dataset after application of exclusion criteria (see Methods). Note: for ‘Practiced years of spiritual practices’, multiple answers were possible. With regards to the practice of spiritual practices, *n* = 48 (12.44%) of the participants used the open response option ‘others’ and the most commonly reported responses fell in the categories ‘singing, dancing, arts’ (*n* = 7), ‘sweat lodge’ (*n* = 5), ‘martial arts’ (*n* = 5), ‘Qigong’ (*n* = 5), ‘Santo Daime’ (*n* = 3).

Total		386		
Female		234 (60.62%)		
Male		148 (38.34%)		
Other		2 (0.52%)		
		Mean ± SD		
Age at time of survey		38.08 ± 9.95		
Years of education		15.69 ± 5.59		
Occupation		Continent		
Employee	155 (40.16%)	Europe		279 (72.28%)
Freelancer	165 (42.75%)	North America		68 (17.62%)
Unemployed	23 (5.96%)	South America		10 (2.59%)
Student (university)	27 (6.99%)	Others		27 (6.99%)
Others	13 (3.37%)			
				Mean ± SD
Frequency of spiritual practices		Spiritual Practice n (%) Years		
Daily	162 (41.97%)	Yoga	248 (64.25%)	7.92 ± 8.35
Weekly	132 (34.20%)	Meditation	300 (77.72%)	7.99 ± 8.72
Monthly	43 (11.14%)	Prayers	168 (43.52%)	13.32 ± 14.45
Rarely	37 (9.59%)	Rituals	218 (56.48%)	8.58 ± 9.95
Never	11 (2.85%)	Mindfulness practice	223 (57.77%)	7.93 ± 8.91
		Breathing exercises	220 (56.99%)	7.27 ± 8.34
		Fasting cure	152 (39.38%)	5.12 ± 7.37
		Being silent	120 (31.09%)	6.29 ± 9.46
		Others	35 (9.07%)	11.47 ± 11.07

SD: standard deviation.

To assess the religious or spiritual practices of our study participants, we asked them what practices they applied, how frequent they apply such practices and for how long they have been practicing them. The results summarised in Table 1 indicate relatively high amounts of diverse spiritual practices. Participants rated the importance of their practices on a scale from ‘0 = not at all‘ to ‘100 = extraordinarily‘ as 85.4 ± 20.45%.

To assess if participants use other substances or ritual plants besides Kambô, we assessed lifetime consumption (summarised in Supplementary Material S2). Reports of substance use (e.g. lysergic acid diethylamide (LSD), cannabis, amphetamine, etc.) do not provide evidence for an association with a particular consumption pattern. However, we found relatively high rates for ayahuasca and Rapé usage in our sample.

We found the mean age of first Kambô usage to be 36.10 ± 10.17 years. Most participants have had more than one Kambô experience, where for *n* = 316 (81.86%) the last experiences was not longer than 12 months ago (see Table 2). When assessing the surroundings in which Kambô was used, our participants reported mainly private settings and to have used it at healing places/temple with a natural healer (see Table 2).

**Table 2.** Previous experiences with Kambô. Multiple answers (yes/no) were possible for the surroundings/setting. Responses for surrounding of previous Kambô usage were given on pre-defined categories. Using the ‘others’ response option, four participants described a setting ‘with a shaman’.

		Mean ± SD	
Age of first Kambô experience		36.09 ± 10.17	
Number of Kambô experiences		Last Kambô experience	
1×	80 (20.78%)	<1 week ago	40 (10.39%)
2–4×	161 (41.82%)	1 week–30 days ago	83 (21.56%)
5–9×	59 (15.32%)	1–12 months ago	193 (50.13%)
10–19×	41 (10.65%)	1–5 years ago	67 (17.40%)
20–35×	24 (6.23%)	>5 years ago	2 (0.52%)
>35×	20 (5.19%)		
Surroundings/setting of previous Kambô experiences			
At home			94 (24.35%)
At a friend’s place/private space			95 (24.61%)
With a natural health professional/healer			125 (32.38%)
At a healing place/temple			105 (27.20%)
In nature			59 (15.28%)
In the Amazon rainforest			33 (8.55%)
At a spiritual festival/healing festival			12 (3.11%)
Within a ceremony with ayahuasca or other psychedelic ritual plants (e.g. peyote, San Pedro, mushrooms, etc.)			70 (18.13%)

SD: standard deviation.

### Motivation for and subjective benefits of Kambô usage

Testing for a broad range of pre-defined personal reasons for using Kambô, the highest report rates were found for ‘desire for general healing’, ‘physical detoxification’, and ‘Improvement of overall well-being’. Among the motives that were treatments of clinically defined symptoms, we found the highest response rates for ‘physical weakness and fatigue/chronic fatigue syndrome’, ‘depression’, ‘weakened immune system’, ‘emotional trauma (e.g. due to abuse)’, ‘substance use disorder/addiction’ and ‘anxiety disorder/panic disorder’. The full report is given in Table 3.

**Table 3.** Personal motives for Kambô treatment. For the personal reasons multiple answers were possible. Responses given for the option ‘others’ were integrated into the predefined categories, where possible. The following additional motives were reported: ‘headaches’ (*n* = 5), ‘preparation for ayahuasca’ (*n* = 4), ‘allergies’ (*n* = 4), ‘improvement of immune system’ (*n* = 2), ‘procrastination’ (*n* = 2), ‘personal growth’ (*n* = 2), ‘healing the heart‘ (*n* = 2), and the following motives mentioned by n = 1 each: ‘benign pituitary tumor’, ‘fibromyalgia’, ‘chorea Huntington’, ‘fertility’, ‘period pain’, ‘asthma’, ‘skin problems’, minimal change disease (MCD)’, ‘obesity’, ‘chronic open wound on the foot’, ‘clarity’, ‘to get rid of fear in general, the fear of life’, ‘overcoming creative blockage’, ‘to support a relative’, ‘increase energy/neuroplasticity’, ‘become a practitioner of this sacred medicine’, ‘intergenerational healing’, ‘following the frogs call’.

Personal reasons for Kambô treatment/usage	
Desire for general healing	65.03%
Physical detoxification	62.18%
Improvement of overall well-being	61.14%
Interest in spiritual experience/spiritual growth	58.55%
Mental/emotional purification	52.59%
Desire to connect with the spirit of the frog	40.16%
Improvement of overall performance	39.64%
Improvement of concentration	35.23%
Physical weakness and fatigue/chronic fatigue syndrome	34.72%
Emotional imbalance	34.20%
Curiosity/interest in extreme experiences	33.94%
Depression	31.87%
Weakened immune system	26.94%
Negative energies from other people	24.09%
Interest in ‘altered states of consciousness‘	23.58%
Improvement of the senses	22.80%
Emotional trauma (e.g. due to abuse)	21.24%
Family or relationship problems	19.95%
Substance use disorder/addiction	19.43%
Anxiety disorder/panic disorder	17.62%
Infections of all kinds (viral/bacterial/fungal, e.g. candida)	17.36%
Gastrointestinal disorders	16.58%
Chronic inflammations	15.28%
Detoxification after a period of using recreational drugs	14.77%
Other mental or emotional problems	14.77%
Chronic pain	14.51%
Autoimmune disease in general	13.21%
Musculoskeletal system issues/back pain (e.g. intervertebral discs)	13.21%
Negative entity attachment	10.36%
Grief (enduring, pathological)	10.10%
Being part of a group in which everyone was receiving Kambô	9.84%
Intestinal or other parasites	8.81%
Impulsive behaviors	8.29%
Thyroid disease	6.74%
Urogenital disorders	4.66%
Lyme disease	4.66%
Sexual dysfunction	4.40%
Manic-depressive disorder	3.89%
Neurodermatitis	3.37%
Rheumatological disorders	3.11%
High blood pressure/low blood pressure	2.59%
Any kind of cancer	2.59%
Obsessive-compulsive disorder (OCD)	1.55%
HIV/AIDS	1.04%
Psychosis or schizophrenia	0.78%
Hepatitis	0.52%
Alzheimer disease	0.26%
Diabetes	0.26%
Multiple sclerosis	0.26%
Parkinson disease	0.00%
Others	16.58%

When asked for the most important reason for using Kambô for the first time, the given open responses fell in the following categories: ‘physical health’ (*n* = 97), ‘detoxification’ (*n* = 85), ‘mental health’ (*n* = 49), ‘interest/curiosity’ (*n* = 36), ‘spiritual growth/healing/altered state of consciousness/finding oneself/the meaning of life’ (*n* = 21), ‘general healing’ (*n* = 17), ‘within a ceremony/preparation for ayahuasca or other ritual plants‘ (*n* = 12), ‘addiction’ (*n* = 10), ‘to connect with the spirit of the frog/follow the frog’s call’ (*n* = 8), ‘to increase strength/energy/vitality/overall performance‘ (*n* = 7), peer pressure/with friends (*n* = 7), ‘relationship issues‘ (*n* = 5), ‘to change my life’ (*n* = 4), 'felt it was right ‘ (*n* = 3).

**Figure 2.**
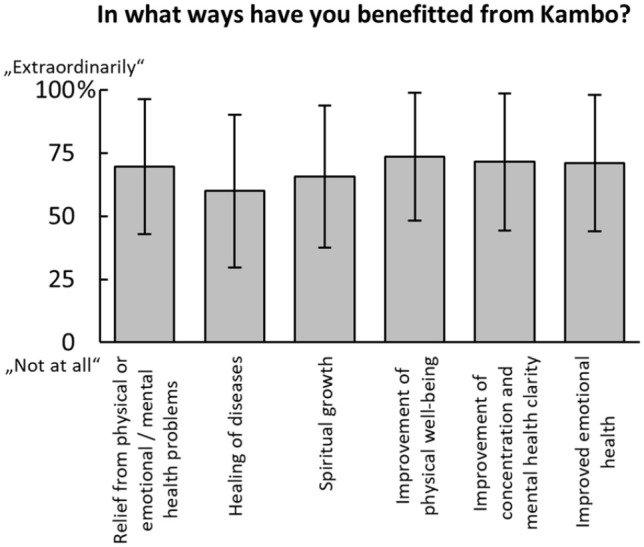
Subjectively reported benefits from Kambô. Participants were asked in what ways they had benefitted from Kambô. Responses on a visual analogue scale (anchored: 0: ‘Not at all’ to 100: ‘Extraordinarily’) on six predefined categories. Bars show mean ± standard deviation (SD).

Next, participants were asked how much they were convinced before and after their first Kambô treatment that it had had a positive effect for them on a scale from ‘0 = not at all’ to ‘100 = fully convinced’. Participants rated that this conviction increased from 70.55 ± 27.70% before to 85.05 ± 23.01% the days after their first Kambô use. In line with this increase, participants rated with 81.35 ± 23.85% (scale from ‘0 = not at all’ to ‘100 = my expectations were exceeded’) that their expectations for Kambô had been met after their first treatment. Please note, that both ratings were given in retrospect, which might have biased these responses. Participants further rated on six pre-defined categories in which regards they had subjectively benefited; results are shown in Figure 2.

### Report of an exemplary Kambô session

In the next part of the survey, participants reported on an exemplary Kambô session. When asking participants to rate how long ago this session was with predefined categories, *n* = 21 (5.44%) reported ‘less than one week ago’, *n* = 43 (11.14%) ‘1 week or more ago’, *n* = 159 (41.19%) ‘1 month or more ago’, *n* = 149 (38.60%) ‘1 year or more ago’ and *n* = 14 (3.63%) ‘5 years or more ago’.

The diversity of surroundings/settings of this session appears comparable to those reported for previous experiences (see above), *n* = 37 (9.59%) reported about a session ‘at home’; *n* = 92 (23.83%) ‘at a friend’s place/private space’; *n* = 138 (35.75%) ‘with a natural health professional/healer’; *n* = 114 (29.53%) ‘at a healing place/temple’; *n* = 41 (10.62%) ‘in nature’; *n* = 13 (3.37%) ‘in the Amazon rainforest’; *n* = 7 (1.81%) ‘at a spiritual festival/healing festival’; *n* = 38 (9.84%) ‘within a ceremony with ayahuasca or other psychedelic ritual plants (e.g. peyote, San Pedro, mushrooms, etc.)’. Via the ‘others’ open response option, *n* = 3 reported ‘in the shamans/practitioners private space’ and *n* = 3 ‘at a Kambô training/retreat’.

In the exemplary session, Kambô had been applied by an ‘Indigenous shaman/medicine man’ for *n* = 52 (13.47%); by a ‘Western shaman/healer/natural health professional’ for *n* = 286 (74.09%); by ‘a person without any special shamanic/healing training (layman)’ for *n* = 27 (6.99%); and ‘I gave it to myself’ was reported by *n* = 15 (3.89%) of participants. For this session *n* = 126 (32.64%) of participants reported that they were the only client; *n* = 243 (62.95%) that they were in a group (group size of 5.71 ± 4.79); and only *n* = 14 (3.46%) that they were alone and applied Kambô themselves.

Before using Kambô, typically a diet or other type of preparation for the Kambô session is performed. When asked how our participants prepared for the Kambô session only *n* = 30 (7.77%) of the survey participants reported that they did not prepare for this exemplary Kambô session; *n* = 252 (65.28%) reported having ‘defined a certain purpose, intention or goal beforehand’; *n* = 243 (62.95%) kept a ‘specific preparation diet’; *n* = 164 (42.49%) reported ‘preparation meeting/conversation with the person who gave the Kambô treatment’; *n* = 121 (31.35%) ‘meditation’; *n* = 47 (12.18%) ‘yoga’; and *n* = 60 (15.54%) ‘Other spiritual practices’. Through the open response option, participants reported the following ‘other’ preparations: ‘fasting’ (*n* = 18), ‘drinking a lot of water’ (*n* = 13), ‘ayahuasca’ (*n* = 6), ‘abstinence of substances’ (*n* = 3). Of the participants, *n* = 279 (72.28%) reported that they changed their nutritional practices or intake of recreational drugs in preparation for the exemplary session. In particular *n* = 201 (52.07%) reported a reduction/dispensation of ‘meat’ consumption; *n* = 173 (44.82%) of ‘all kinds of animal products’; *n* = 87 (22.54%) ‘fats/oils’; *n* = 205 (53.11%) ‘refined sugar’; *n* = 49 (12.69%) ‘carbohydrates’; *n* = 22 (5.70%) ‘proteins’; *n* = 245 (63.47%) ‘alcohol’; *n* = 145 (37.56%) tobacco/nicotine; *n* = 183 (47.41%) ‘caffeine’; *n* = 192 (49.74%) ‘recreational drugs’. As open responses for ‘other’, participants reported reduction/dispensation of ‘salt/spices’ (*n* = 10) and ‘(citrus) fruits’ (*n* = 3).

When asking for consumption of ritual plants within 30 days before the Kambô experience (open response format), the use of ayahuasca/Daime/Yagé was highest with *n* = 89, confirming the previously reported association between ayahuasca and Kambô usage; *n* = 16 reported Peyote/San Pedro; *n* = 16 mushrooms; *n* = 3 LSD. With regards to ritual plant usage, *n* = 50 reported Rapé/Mapacho and *n* = 14 Sananga. The following recreational and/or addictive substances were reported: *n* = 52 cannabis; *n* = 21 tobacco (ritual/cigarettes); *n* = 12 alcohol; *n* = 6 ecstasy, molly; *n* = 5 speed; *n* = 3 coca leaves; *n* = 3 cocaine (powder). However, our data do not permit to draw conclusions on the framework in which the combination of ritual plants with Kambô was conceptualised by the participants in our sample, e.g. we do not know about the temporal association of the application of both substances or about which specific function they were believed to fulfil in the ritual.

The average amount of ‘dots’ that were applied in this exemplary Kambô session were 6.3 ± 3.3 (mean ± SD), with a range from 1 to 20 dots. The acute process was reported to have had a duration of less than 15 min for *n* = 39 (10.10%); 15 min or more for *n* = 149 (38.60%); 30 min or more for *n* = 137 (35.49%); 60 min or more for *n* = 35 (9.07%) and 90 min or more for *n* = 24 (6.22%).

To test if the duration of an acute experience directly relates to the number of dots, we tested for their correlation (Spearman), which was not significant (*r*_s_ = 0.0884, *p* = 0.0845, *n* = 382).

We asked participants to report the occurrence of a pre-defined set of possible symptoms during the acute Kambô experience and found, on the one hand, effects that are considered aversive, but, on the other hand, also positive subjective experiences (Table 4). Most participants reported ‘vomiting’, ‘hot flashes/feeling of fever’, ‘nausea’, ‘racing heart’, ‘sweating’ as physiological symptoms. Fewer participants reported the occurrence of psychological problems. Interestingly, the most common psychological symptom reported (*n* = 162, 41.97%) was ‘feeling that the spirit of the frog is with me/present in myself/integrated in my being’. Also notable is that *n* = 66 (17.10%) of participants reported ‘anxiety/panic’ opposed to *n* = 70 (18.13%) reporting ‘joy’.

**Table 4.** Acute effects/symptoms during exemplary Kambô experience. Percentage of participants who reported to have experienced the listed symptoms. Note: Multiple answers were possible. Using the open response option ‘others’, the following additional acute symptoms were reported: ‘hallucinations’ (*n* = 6), ‘relief/release’ (*n* = 6), ‘sensory disturbance’ (*n* = 6), ‘cramps’ (*n* = 6), ‘sadness’ (*n* = 4), ‘weakness’ (*n* = 4).

Acute physiological symptoms	
Vomiting	86.53%
Hot flashes/feeling of fever	73.06%
Nausea	68.13%
Racing heart	67.10%
Sweating	59.07%
Swelling of the face	55.18%
Diarrhoea	41.19%
Dizziness	34.97%
Chills	29.02%
Stomach pains	25.91%
Swelling of the throat	24.35%
Tremors	23.58%
Abdominal pain	20.21%
Shortness of breath	18.39%
Skin changes	16.58%
Whole-body pain	13.47%
Headache	12.18%
Loss of consciousness	12.18%
Swelling of the whole body	8.55%
Increased urination	6.74%
Cough	5.96%
Back pain	4.92%
Swelling of arms/legs	4.66%
Itching	4.40%
Chest pains	3.63%
Swelling of the trunk	2.33%
Acute psychological symptoms	
Feeling that the spirit of the frog is with me/present in myself/integrated in my being	41.97%
Joy	18.13%
Anxiety/panic	17.10%
Feeling of oneness with everything	14.77%
Desperation	11.14%
Fear of death	9.07%

**Figure 3.**
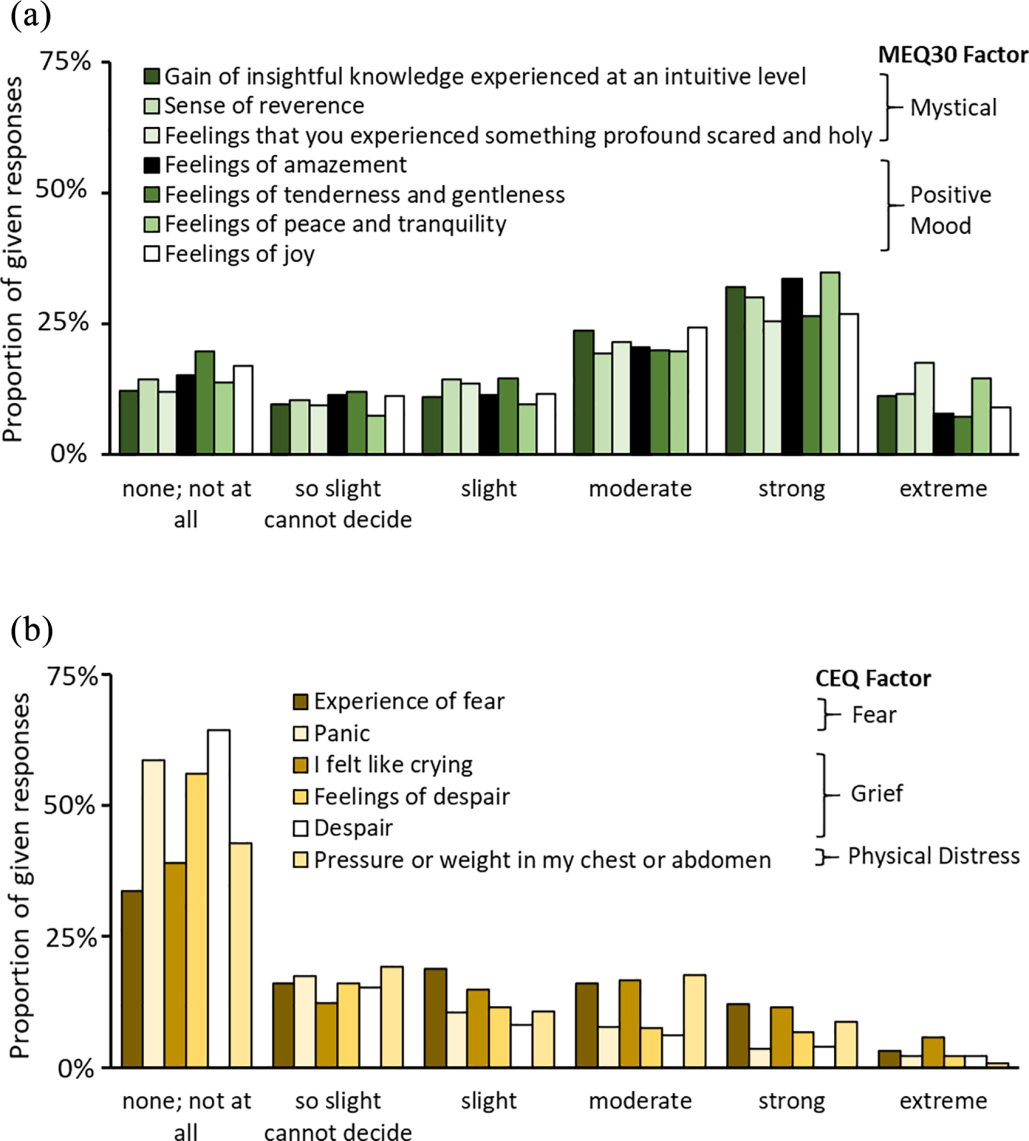
Acute effects of Kambô: (a) participants reported aspects of mystical experiences throughout seven pre-selected items of the Mystical Experience Questionnaire (MEQ), with ratings of moderate to strong effects for most items and (b) on the contrary, aspects of challenging experiences, assessed with six pre-selected items of the Challenging Experience Questionnaire (CEQ) were rather rare. Items were pre-selected from the questionnaires with suggestions from a Kambô practitioner.

To further characterise our participants’ acute subjective experiences, they rated items of the MEQ and CEQ for which results are presented in Figure 3. When participants were additionally asked if they felt that Kambô induced an altered state of consciousness, participants rated on a scale from 0 = ‘not at all’ to 100 = ‘profoundly’ with 55.95 ± 32.69% (mean ± SD).

When testing for a relationship between the applied Kambô dosage, we found a significant positive correlation between dosage (number of dots) and the MEQ item ‘Feeling that you experienced something profoundly sacred and holy’, *r*_s_ = 0.1604, *p* = 0.0031 (Only data sets were included where all MEQ and CEQ items were answered, resulting in *n* = 338 data points). No other correlations of the MEQ and CEQ items were significant (*p* < 0.05 Bonferroni corrected for seven comparisons 0.05/7 = 0.0071).

### Long-term changes and effects of a Kambô treatment

In the last part of the survey, we assessed long-term changes of physical and mental or emotional health issues by asking for the emergence of new issues and the intensification or deterioration of existing issues.

When asking for the emergence of any new physical health issues in the days after the Kambô treatment, *n* = 30 (7.77%) answered ‘yes’, *n* = 340 (88.08%) answered ‘no’ and *n* = 16 (4.15%) answered ‘don’t know’. The following new problems were reported: ‘weakness/exhaustion/fatigue’ (*n* = 6), ‘cold/fever’ (*n* = 5), ‘pain’ (*n* = 5), ‘increased sweating/night sweats’ (*n* = 2), ‘swelling’ (*n* = 2), ‘arthritis’ (*n* = 2), ‘neurodermatitis’ (*n* = 1), ‘amnestic aphasia’ (*n* = 1), ‘deterioration of eyesight’ (*n* = 1), ‘diarrhoea’ (*n* = 1), ‘stronger menstruation’ (*n* = 1), ‘skin irritation’ (*n* = 1), ‘vaginal infection’ (*n* = 1), ‘stiff neck’ (*n* = 1), ‘allergies’ (*n* = 1), ‘shingles’ (*n* = 1). Of those participants *n* = 9 (2.33%) reported, that these physical problems were so strong and long-lasting that they saw a doctor. The reported symptoms of these nine participants were ‘weakness/exhaustion/fatigue’, ‘pain’, ‘arthritis’, ‘neurodermatitis’, ‘stronger menstruation’, ‘cold/fever’.

When asking participants if any previously existing physical health issues became more severe or re-emerged in the days after the Kambô treatment, *n* = 38 (9.84%) answered ‘yes’, *n* = 327 (84.72%) ‘no’ and *n* = 14 (3.63%) ‘don’t know’, *n* = 7 did not answer the item. Participants indicated the following physical health issues: ‘muscular pains/tensions/cramps’ (*n* = 6), ‘neurodermatitis/eczema’ (*n* = 6), ‘weakened immune system/susceptibility to infection’ (*n* = 4), ‘arthritis/joint pains’ (*n* = 3), ‘weakness/fatigue’ (*n* = 2), ‘migraine’ (*n* = 2), ‘shingles’ (*n* = 2), ‘toothache/root infection’ (*n* = 2), ‘asthma’ (*n* = 1), ‘swollen lymph nodes’ (*n* = 1), ‘swollen face’ (*n* = 1), ‘candida’ (*n* = 1), ‘premenstrual syndrome’ (*n* = 1), ‘increased sensitivity (increased sense of smell)’ (*n* = 1), ‘chronic inflammation’ (*n* = 1), ‘dizziness’ (*n* = 1). Of those, *n* = 5 (1.30%) indicated that these symptoms were so strong and long-lasting that they saw a doctor for the following symptoms: ‘neurodermatitis/eczema’, ‘weakened immune system’, ‘back and neck pain’.

When asking if participants felt that Kambô had ever led to a long-lasting deterioration of physical health issues, *n* = 11 (2.85%) answered ‘yes’, *n* = 360 (93.26%) answered ‘no’ and *n* = 13 (3.37%) answered ‘don’t know’ (no report of specific issues was collected).

When asking for the emergence of any new mental or emotional problems in the days after the their Kambô experience, *n* = 17 (4.40%) answered ‘yes’, *n* = 348 (90.16%) answered ‘no’ and *n* = 8 (2.07%) answered ‘don’t know’, *n* = 13 did not answer the question. The following issues were reported: ‘sadness, depression, lack of drive’ (*n* = 6), ‘psychosis’ (*n* = 2), ‘suicidality’ (*n* = 1), ‘bad energies’ (*n* = 1), ‘emotional instability’ (*n* = 1), ‘loss of self’ (*n* = 1), ‘substance use’ (*n* = 1), ‘insomnia’ (*n* = 1). Five participants (1.30%) reported that they saw a doctor because of these new mental or emotional issues, where the following symptoms were reported: ‘psychosis’, ‘loss of self’, ‘substance use’.

When asking participants if any previously existing mental or emotional health issues become more severe or re-emerged in the days after their Kambô experience, *n* = 26 (6.74%) answered ‘yes’, *n* = 344 (89.12%) ‘no’ and *n* = 10 (2.59%) ‘don’t know’, *n* = 6 did not answer the item. Participants reported the following mental or emotional health issues: ‘depression’ (*n* = 7), ‘anxiety/panic/insecurity’ (*n* = 8), ‘grief’ (*n* = 5), ‘emotional issues’ (*n* = 3), ‘addiction’ (*n* = 4), ‘anger’ (*n* = 2), ‘obsessive-compulsive disorder’ (*n* = 1), ‘relationship issues’ (*n* = 1), ‘loneliness’ (*n* = 1), ‘disorientation’ (*n* = 1), ‘insomnia’ (*n* = 1). Nine participants (2.33%) indicated, that these symptoms were so strong and long-lasting that they saw a doctor with the following symptoms: ‘anxiety/panic/insecurity’, ‘depression’, ‘emotional issues’, ‘addiction’, ‘obsessive-compulsive disorder’.

When asking if participants felt that Kambô had ever led to a long-lasting deterioration of mental or emotional health issues, *n* = 7 (1.81%) indicated ‘yes’, *n* = 368 (95.34%) answered ‘no’ and *n* = 9 (2.33%) indicated ‘don`t know’ (no report of specific issues was collected).

When asking participants if they overall ever regretted using Kambô, *n* = 14 (3.63%) responded with ‘yes’, *n* = 362 (93.78%) with ‘no’, *n* = 8 (2.07%) with ‘don’t know’ and *n* = 2 (0.52%) gave no response. On the contrary, when asked to what extent one’s life had been influenced in a lasting and profound way by the use of Kambô, participants rated 74.43 ± 28.56% (on a scale from 0 = ‘not at all’ to 100 = ‘extraordinarily’).

**Figure 4.**
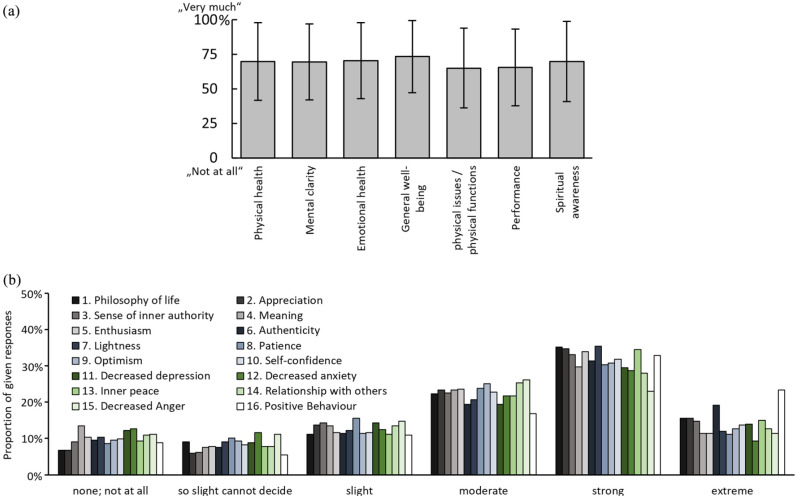
Subjective experience of positive long-lasting effects: (a) positive long-term effects were assessed on seven pre-defined categories, rated on a visual analogue scale (anchored: 0: ‘not at all’ to 100: ‘very much’) and (b) ratings of long-term effects by means of ratings of PEQ items on a six-point scale (from ‘none’ to ‘extreme’), presented as proportion (percentage) of given responses. The full formulations of items was as follows: (1) the experience has changed your philosophy of life positively; (2) your appreciation for life has increased; (3) you have a greater sense of inner authority in your life; (4) your life has more meaning; (5) you have more enthusiasm for life in general; (6) you are a more authentic person; (7) you have more good natured humour/playfulness/lightness; (8) you have more patience/ability to tolerate frustration; (9) you have more optimism; (10) your self-confidence/self-assurance has increased; (11) feelings of depression have decreased; (12) feelings of anxiety have decreased; (13) you have more inner peace (i.e. centredness, serenity, calmness); (14) you have a more positive relationship with others; (15) your negative expression of anger (e.g. ridicule, outward expression of irritability toward others) has decreased.

We asked participants for how long they experienced long-term effects of their exemplary Kambô session and these were reported to have lasted: ‘up to 3 days’ *n* = 33 (8.55%); ‘4–7 days’ *n* = 46 (11.92%); ‘8–30 days’ *n* = 95 (24.61%); ‘1–6 months’ *n* = 101 (26.17%); ‘7–12 month’ *n* = 23 (5.96%); more than 1 year’ *n* = 11 (2.85%); ‘ the effects are noticeable after more than 1 year and are still there’ *n* = 49 (12.69%); ‘I don’t remember’ *n* = 26 (6.74%). Participants reported what positive long-lasting effects they experienced (on a scale from 0 = ‘not at all’ to 100 = ‘very much’), as displayed in Figure 4.

In the initial assessment of substance usage, participants were asked if their frequency of consumption has changed due to their Kambô experience. Participants reported: *n* = 64 (16.58%) ‘never used since then’; *n* = 193 (50.00%) ‘less frequent since then’; *n* = 4 (1.04%) ‘more frequent since then’; *n* = 104 (26.94%) ‘unchanged since the Kambô treatment(s)’; *n* = 15 (3.89%) ‘I don’t have any experiences with these substances’. When asking explicitly if participants changed their intake of foodstuffs or medication, alcohol, tobacco, caffeine or other recreational drugs, *n* = 286 (74.09%) participants responded with ‘yes’ (‘no’: *n* = 99; no response: *n* = 1). Consecutive reports on the reduction are summarised in Table 5. Of those participants (*n* = 75) who reported ‘substance use disorder/addiction‘ as a motivation, 28.00% (*n* = 21) reported that they never used the substance since then and 64.00% (*n* = 48) that they consumed the substance less frequently. It should be noted that due to the relatively small number of cases, no analysis of substance classes was conducted.

**Table 5.** Reduction of foodstuffs, medication, alcohol, tobacco, caffeine or other recreational drugs after the use of Kambô.

	1–7 days		8–30 days		>30 days, but not permanently		Permanent		No changes	
	*n*	%	*n*	%	*n*	%	*n*	%	*n*	%
Meat	16	4.15	32	8.29	44	11.40	95	24.61	79	20.47
Alcohol	21	5.44	34	8.81	71	18.39	107	27.72	34	8.81
Tobacco/nicotine	23	5.96	16	4.15	37	9.59	82	21.24	89	23.06
Medication	7	1.81	7	1.81	29	7.51	85	22.02	105	27.20
Recreational drugs	9	2.33	23	5.96	47	12.18	89	23.06	80	20.73
All kinds of animal products	18	4.66	30	7.77	51	13.21	49	12.69	100	25.91
Fats/oils	16	4.15	28	7.25	26	6.74	21	5.44	140	36.27
Raffinated sugar	26	6.74	37	9.59	49	12.69	76	19.69	69	17.88
Carbohydrates	22	5.70	22	5.70	32	8.29	25	6.48	128	33.16
Proteins	18	4.66	14	3.63	15	3.89	14	3.63	160	41.45
Caffeine	30	7.77	27	6.99	38	9.84	56	14.51	96	24.87

**Figure 5.**
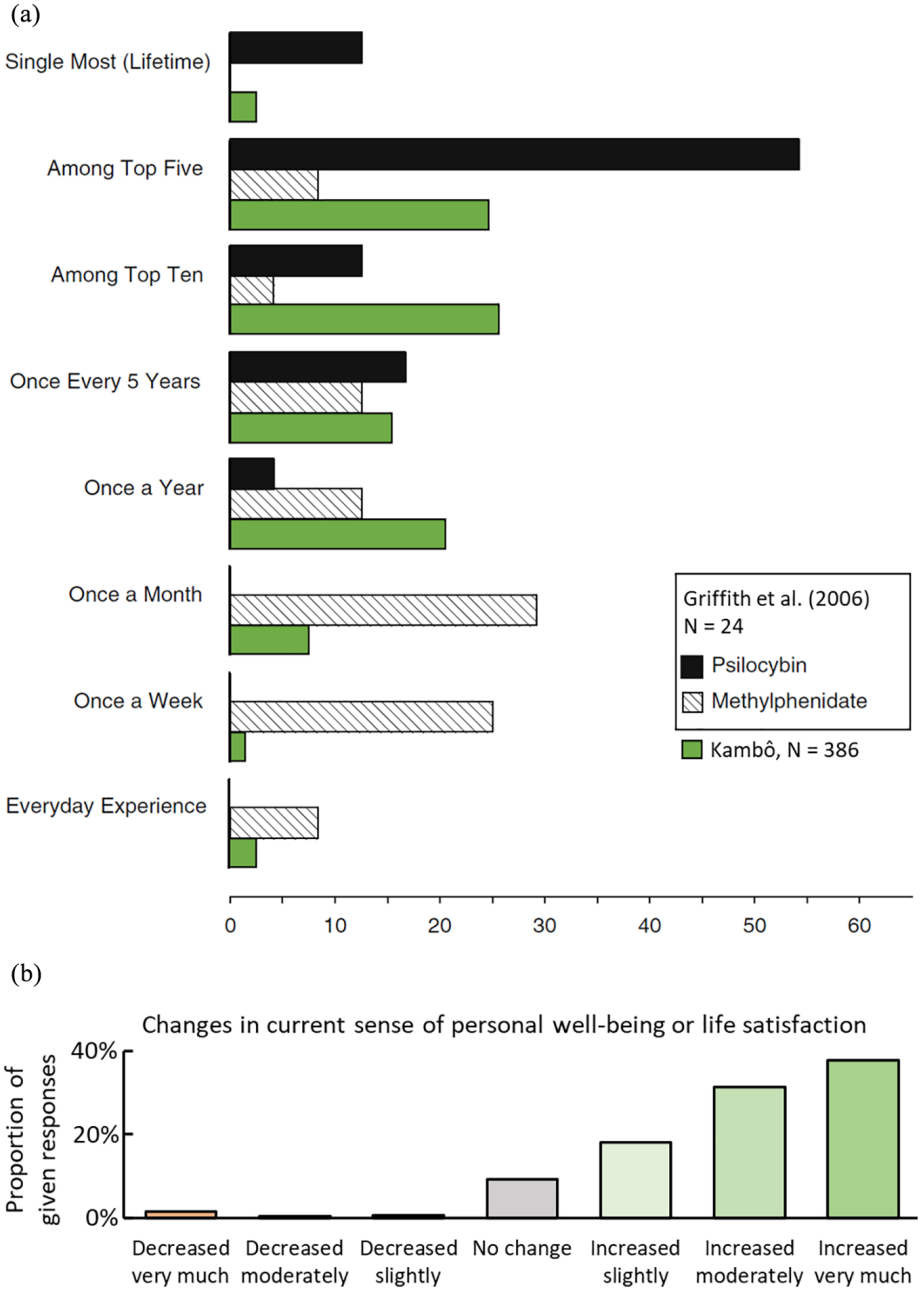
Meaningfulness and subjective effects on well-being and life satisfaction: (a) comparison to the retrospective assessment of the effects of psilocybin; figure adapted from Griffiths et al. (2006), which was a randomised controlled study (*n* = 24) in which effects of psilocybin (black column) were compared to methylphenidate (grey column) with regard to how experiences were retrospectively appraised by participants. Participants were asked how personally meaningful the experience had been when compared to everyday experiences, experiences which occur once a week, once a month, once a year, once every 5 years, top 10 or top five experiences of their lives, single most (lifetime) personally meaningful experience of their lives. Data from our study were added regarding exemplary Kambô session (green column). (b) Subjective rating on the long-term effects on current sense of personal well-being and life satisfaction. Participants rated from ‘decreased very much’ to ‘increased very much’.

Finally, we were interested in participants’ evaluation of the relevance of their Kambô experience for their lives. In Figure 5(a) the ratings for how personally meaningful participants rated the Kambô experiences are presented including comparison data from psilocybin and methylphenidate by Griffiths et al. (2006). When asked about the degree of spiritual significance, 3.89% (*n* = 15) reported ‘not at all’; 8.81% (*n* = 34) ‘slightly’; 23.06% (*n* = 89) ‘moderately’; 34.46% (*n* = 133) ‘very much’; 25.65% (*n* = 99) ‘among the five most spiritual significant experiences of my life’; and 4.15% (*n* = 16) ‘the most spiritual significant experience of my life’. When asked if they believed that the Kambô experience and contemplation of that experience induced changes, 87.31% of participants reported an increase in their current sense of personal well-being or life satisfaction (Figure 5(b)).

## Discussion

To our knowledge, this is the first study investigating the use of Kambô in Western countries. Participants were mostly well-educated individuals with a high overall interest in spiritual practices, with an elevated level of the use of ayahuasca and other ritual plants. Users reported a plethora of different motivations for using Kambô with salutogenic aspects regarding physical and mental health being dominant. Kambô was received predominantly in a group of several recipients in neo-shamanic settings. Dose-response relationship remained inconclusive. Acute effects included severe physical reactions and mild psychoactive effects, whereas intensive and predominantly positive subacute and long-term effects towards more health-oriented behaviours were reported. Few participants reported persisting problems of physical or mental health which they attributed to Kambô. Notably, the majority of participants stated that Kambô had been of a high personal significance for their lives.

### Participant characteristics

We were interested to investigate whether the use of Kambô was associated with certain participant characteristics and with the use of recreational or addictive substances. We found that participants were mostly well-educated individuals with a high amount of self-employed workers and freelancers. Participants displayed a mean age for first using Kambô of 36.1 years and a high overall interest in spiritual practices, not restricted to any specific religious background. Lifetime prevalence of the use of alcohol and other recreational drugs displayed somewhat higher levels than in Western countries’ general populations (Mounteney et al., 2016). Of note, our data corroborate a certain overlap between the usage of Kambô and other nature-derived substances used in ritual contexts, including, above all, ayahuasca (67.88%), Rapé tobacco (77.98%) and Sananga eye drops (61.66%). Notably, Rapé tobacco and Sananga are ritual plants that are often associated with the use of ayahuasca, but are widely unknown to Western populations outside of that context (Oliveira, 2019). Furthermore, our data suggest that Kambô was often administered after a recent intake of ayahuasca, even if it was predominantly not used during the same ceremony. Moreover, other lifetime use of nature-derived psychedelics like peyote, psilocybin, N,N-dimethyltryptamine and cannabis has also been relatively high among our participants when compared to general populations. However, the pattern of previously consumed substances indicates that Kambô users would not be characterised by regular recreational drug use, but rather belong to a group of spiritual seekers using psychedelic substances for self-exploration. In conclusion, the associations between the use of Kambô and the use of ritual plants like ayahuasca, which have been reported for Amazonian ethnicities and for urban centres in Brazil (De Lima and Labate, 2008), also appear to apply to the Kambô using subcultures and neo-shamanic frameworks in other Western countries. Originally, it appears that some Amazonian cultures like the Kaxinawa that use Kambô also use ayahuasca (Hesselink, 2018b), but we are not aware of any scientific work stating that both techniques are combined. As noted above, it seems that Kambô is usually carried out for purposes that differ from ayahuasca uses, and that it can be applied by non-shamans, too, whereas ayahuasca is mostly restricted to shamanic use (Hesselink, 2018b). As Labate and De Lima (2015) pointed out, the ‘shamanization’ of Kambô will have taken place only after Kambô had left the Amazonian rain forest. Of note, our data do not permit to draw conclusions on conceptual frameworks in which Kambô and ritual plants are combined in our ‘Western’ sample, which would be interesting to investigate in further studies.

### Motivations for using Kambô

One of the pivotal questions of our study was to investigate the participants’ motivations for using Kambô. Participants reported a broad variety of reasons and aims for using Kambô, partly reflecting promises of a panacea to heal almost any existing pathology. General motives involving salutogenic aspects (‘desire for general healing’) were most common, together with the idea of ‘detoxification’ which appears to be one of the core objectives of Kambô rituals (De Lima and Labate, 2008; Den Brave et al., 2014; Gorman, 1993). Given a magical and holistic system of reference, it makes sense that users seek for salutogenic aspects, general healing and improvement of well-being, aside from treating specific pathologies. Accordingly, motivations were commonly associated with physical, psychological and spiritual aspects of detoxification and symptoms of overcoming various types of ‘weakness’, partly reflecting expectations regarding stimulating effects of Kambô and corresponding Amazonian motivations of use in the above mentioned sense of a cure against ‘panema’ (Labate and De Lima, 2015). With regard to psychiatric conditions, overcoming symptoms of depression, substance use disorders and anxiety disorders were among the most commonly reported reasons for using Kambô, which on the one hand may partly reflect the relative overall prevalence of these problems in psychiatric populations. On the other hand, depression, addiction and anxiety may show certain aspects of so perceived ‘weakness’, making attractive a treatment which holds the promise to purify from ‘negative energies’ or other obstacles, i.e. an ‘antidote for panema’ (De Lima and Labate, 2008). In summary, spiritual growth, salutogenesis and healing specific pathologies, and spiritual seeking were reported as most common motivations for using Kambô in our sample.

### Preparation and settings

We also wanted to find out if and how participants prepared for Kambô sessions and how specific settings were arranged in which the sessions took place. Most participants reported specific preparations. Most common was a specific diet prior to the session and the definition of a specific intention for the session. Drinking large amounts of water before receiving Kambô is considered as an integral part of the cleansing ritual by many practitioners (personal communication, German practitioner). In fact, case reports exist where water intoxication due to an to huge amount had occurred, while it is unclear which measures are undertaken by practitioners to avoid water intoxication (Agüero-González et al., 2019; Campodónico et al., 2019; Leban et al., 2016). In the survey at hand, we did not ask explicitly for water consumption as part of the Kambô treatment. Thirteen participants used the open response possibility to say that they drank lots of water, while this number is likely to underrepresent the real amount of participants who did so, as filling in an open response field requires special effort. Almost three out of four participants reported that they had received Kambô at a healing place or temple, from a ‘Western shaman’, healer or health professional, indicating that the majority of our participants stemmed from urban centres, using neo-shamanic adaptations of the Kambô ritual. Only very few participants (3.46%) stated that they had applied Kambô to themselves, corroborating previous reports that the substance is almost always received by a second person, dividing between applicators and recipients (De Lima and Labate, 2008). In addition, 62.95% of participants stated that they had received Kambô in a group setting when compared to 32.64% who had been the session’s only recipient. Notably, our data do not permit to draw conclusions about the ritual or therapeutic framework in which Kambô sessions have taken place. Therefore, additional studies are needed to learn more about the concepts of Western Kambô practitioners.

### Acute effects of Kambô

In addition, our study focused on the user's subjective perception of the acute effects of Kambô during the session. Almost three-quarters of participants stated that the acute process had a duration of between 15–60 min, with only about 15% exceeding that timeframe. Reported effects included strong physiological reactions (vomiting, hot flashes, feeling of fever, nausea, racing heart, sweating), whereas psychological effects were reported by fewer participants, corroborating previous anecdotal observations (Daly et al., 1992; Hesselink, 2018c).

We further asked our participants to report psychological reactions to Kambô in a direct and spiritual sense. Interestingly, many users stated that they aimed to ‘connect to the spirit of the frog’, which is in keeping with the notion that the ‘spirit of the frog’ detoxifies by ‘travelling’ through body and psyche, ‘scanning’ and relieving the users from pathogenic psycho-bio-spiritual blockades and obstacles, which has been anecdotally pointed out by researchers (Hesselink and Winkelman, 2019) and a German Kambô practitioner (personal communication). Furthermore, feelings of ‘anxiety’ or ‘joy’ were reported by approximately one-fifth of our participants. Nevertheless, even if a great number of participants described at least some kind of acute psychoactive effects occasioned by Kambô, these were not reported consistently and were not comparable to the intensity and variety of phenomena associated with acute psychedelic experiences (Schmidt and Berkemeyer, 2018). When asked specifically if participants considered the Kambô experience as an altered state of consciousness they provided a rating on a scale from ‘0: not at all’ to ‘100: profoundly’ with 55.95 ± 32.69% (mean ± SD). This corroborates findings from a retrospective pen-and-paper study published elsewhere (Schmidt et al., 2020), where moderate psychoactive effects were reported by users. In literature to date, it has been controversial if Kambô can occasion alterations of consciousness, and if so, the mechanisms of action would remain elusive. On the one hand, the peptide structure of the substances identified in the secretion (Erspamer et al., 1993) make it highly unlikely that any direct pharmacodynamic effects upon the central nervous system are present. On the other hand, visionary phenomena have been reported anecdotally (Gorman, 1993), but it remains unclear whether these might have been facilitated by the simultaneous use of other ritual plants. Overall, previous studies mostly described acute physical phenomena following the application of Kambô which are corroborated by the findings from our study (Daly et al., 1992; Erspamer et al., 1993; Hesselink, 2018b).

In conclusion, our findings do not suggest that Kambô acutely exhibits strong psychoactive effects, which is in line with anecdotal observations from previous ethnological and pharmacological studies. Nevertheless, controlled studies would be needed to completely rule out psychoactive properties.

### Effects and dosage

Regarding acute effects, we also investigated whether effects were associated with dosage, i.e. the number of dots that have been applied. Of note, the average amount of ‘dots’ was 6.3 dots per session, ranging between 1–20 dots. With regard to a dose-response relationship between Kambô and the experienced effects, we did not find any association with the duration of the acute effects, failing to confirm previous observations (Daly et al., 1992). It must be noted that ‘the amount of dots’ is not a standardised measure, and the amount of substance and the quality of the Kambô preparation might be variable. Exploratory testing for correlations with the effects reported in terms of MEQ and CEQ items revealed a positive correlation (*r*_s_ = 0.1604, *p* = 0.0031) between dosage and the ‘feeling that you experienced something profoundly sacred and holy’ only, which is one facet of mystical experiences as typically reported under the influence of psychedelic substances (Griffiths et al., 2006). Conversely, no other associations between dosage and psychological effects were found. This lack of a dose-response relationship could be interpreted in different ways. On the one hand, it might be that the amount of active ingredients in different Kambô preparations is highly variable and therefore the number of dots is not representative of the dosage. On the other hand, it could be interpreted, that the variable surroundings and procedures play an important role for the subjective experience during Kambô, which has more influence than the dosage as such. However, controlled studies would be required to determine if Kambô effects underlie a dose-response relationship.

### Positive and negative subacute and long-term effects on health

Positive effects on physical and mental health have repeatedly been named as motivations for the use of Kambô by both practitioners and recipients, whereas only few reports on medical or psychiatric complications of Kambô use are reported in biomedical literature (for a short review, see (Silva et al., 2019)). Thus, another important focus of our study was to investigate which positive and negative effects were reported by the users. In a previous study, it has anecdotally been stated that sub-acute effects of Kambô include ‘increase in physical strength, heightening of senses, resistance to hunger and thirst, exalted capacity to face stress situations’ (Erspamer et al., 1993).

Generally, more than four out of five participants stated that their expectations had been met after their first treatment, with a high number claiming that their expectations had even been surpassed. As mentioned above, participants were asked to report about one specific session with Kambô which they found exemplary for their overall experiences. Surprisingly, almost all participants claimed that effects of their exemplary Kambô session had by far outlasted the acute effects of the session, with almost one-fifth stating that effects had lasted for more than 6 months, including more than 10% who claimed effects after more than 1 year. Moreover, almost three-quarters of participants stated that Kambô had influenced their lives in a lasting and profound way. It is important to point out, however, that our results will have been affected by a selection bias, given the recruitment of participants via websites from the field, which might have led to over-emphasising positive results.

One interesting finding about beneficial effects included that the majority of participants claimed to have reduced substance use following the use of Kambô, with more than 15% stating that they had ‘never used since then’ and one-half stated that they had reduced consumption. Almost three-quarters stated that they had reduced intake of alcohol, tobacco, caffeine or other recreational drugs. Among the 75 participants who had reported the treatment of substance use disorder as motivation for using Kambô, almost one-third claimed that they had never used that recreational substance again and almost two-thirds reported that they had decreased their use. Again, to date, comparable claims of anti-addictive effects of single-dosage or few times applications have been attributed to hallucinogenic substances only, including ketamine (Krupitsky et al., 2002), LSD (Krebs and Johansen, 2012), psilocybin (Bogenschutz et al., 2015) ayahuasca (Thomas et al., 2013) and ibogaine (Schenberg et al., 2014). Nevertheless, anti-addictive mechanisms of action of each named substance remain speculative, which is also true for the putative effects of Kambô. Interestingly, users did not only report that the use of addictive or recreational substances decreased following Kambô use, but also that general consumption patterns of food and foodstuffs and behavioural patterns had changed. This finding is in keeping with salutogenic aims and intentions, a holistic perspective on body and mind and the connection between humans and natural surroundings (Hesselink and Winkelman, 2019).

When asking participants how spiritually significant their Kambô experiences had been for their lives, we found, surprisingly, that 25.65% of participants stated that Kambô had been among the five most spiritual experiences in their lives, and 4.15% of participants reported that Kambô was the most spiritually significant experience in their lives. Finally, 87.31% participants stated that Kambô had improved their current sense of personal well-being or life satisfaction. Interestingly, this observation on long-term effects somewhat resembles findings on the subjective appraisal of participants who had received a single application of high-dose psilocybin (Griffiths et al., 2006), even if the acute effects of psilocybin in most aspects differ from Kambô (Figure 5(b)).

The observations of our study indicate that, for a majority of users, Kambô showed strong subjective effects associated with a high appraisal and high personal meaning of the experience. However, in our study, improvements in physical and psychological health remain subjective and its proposed mechanisms of action remain mostly hypothetical in nature, including the pivotal question in how far beneficial effects have been associated with expectational or setting biases.

Regarding negative effects, we tried to differentiate between new physical or mental health issues, previously existing physical or mental health issues that had become more severe or re-emerged in the days after Kambô and long-lasting physical or mental health issues to rule out what has been conceptualised as ‘initial worsening’ in complementary medicine. In addition, we also wanted to know in how many cases both had led to help-seeking behavior within the medical healthcare system. Notably, new (7.7%) and previously existing or re-emerging (9.84%) physical health issues did not substantially differ from each other with regard to symptoms or frequency of symptoms. Physical health issues leading to professional help-seeking behavior included mostly unspecific symptoms, above all, associated with weakness and pain. New mental or emotional problems (4.40%) and previously existing mental or emotional health problems becoming more severe or re-emerging (6.74%) most frequently presented as symptoms of depression and anxiety. Accordingly, physical and mental health problems overlap regarding loss of energy, which had been named as motivation for the use of Kambô. As mentioned above, most of these problems appear to have been transient in nature and temporarily restricted to a few days after the use of Kambô, labelled as ‘initial worsening’ as integral part of the cleansing or healing process by practitioners (German practitioner, personal communication). Nevertheless, given the overall high number of participants reporting such an increase of symptoms, more research is needed to investigate possible physical and psychological side effects of Kambô and to rule out genuine health concerns.

In addition to merely transient negative after-effects, 11 participants (2.85%) reported a previously non-existing, long-lasting deterioration of physical health problems, whereas seven participants (1.81%) reported that Kambô had led to long-lasting deterioration of mental or emotional health issues. Overall, 14 participants regretted using Kambô. Although to date no clinical studies have been carried out with Kambô (as it is mostly unknown to Western biomedical literature and probably due to its unknown safety profile), these findings are in accordance with recent case reports suggesting an association of Kambô use with serious adverse effects, including psychosis (Roy et al., 2018), prolonged symptoms of intoxication (Li et al., 2018), a case of sudden death (Aquila et al., 2018), toxic hepatitis (Joanna and Łapiński, 2017), and a case of dermatomyositis (De La Vega et al., 2020) (for a brief summary, see (Silva et al., 2019)). In addition, three case reports have indicated poisoning by drinking excessive amounts of water in the framework of Kambô ceremonies, including a syndrome of inappropriate antidiuretic hormone secretion (SIADH) (Leban et al., 2016), and two cases of severe hyponatraemia (Agüero-González et al., 2019; Campodónico et al., 2019). To our knowledge, to date there are no published cases of Kambô-associated side effects beyond the aforementioned cases. Even if it remains partly unclear whether the described symptoms can causally be attributed to Kambô use in our study and in some of the mentioned cases, there are hardly any data available regarding the risk profile of the *Phyllomedusa* peptides in humans. Moreover, to our knowledge to date no quality standards or safety guidelines are available, e.g. providing information on screening of participants and on which participants should be excluded from a Kambô session. We hypothesise that currently different measures are applied by different practitioners when selecting participants for Kambô sessions. To date, the authors are not aware of any legal regulations with regards to Kambô or its chemical compounds. Given the reported effects, the risk of recreational use or habit-forming characteristics do not appear to be very likely, but further research is needed to better understand subjective effects and mechanisms of action, in order to determine the risk profile of Kambô.

### Limitations

Our findings are not representative of the usage of Kambô across cultures and subcultures. They cannot be generalised regarding use patterns of Kambô, as our recruitment was distributed with support from Kambô-related social media websites, Kambô practitioners, scientists and other individuals related to the field of natural psychedelics or other ritual plants. This might have resulted in a selection bias in our sample and is also reflected in the majority of participants living in Europe. This selection bias might have affected participants’ characteristics, motivations and settings associated with the use of Kambô. However, it appears that given the specifics of the Kambô ritual and its effects, it does not seem very likely to find recreational use or other forms of use that are divergent from those found in our sample, even in heterogenous samples of Western users. Nevertheless, our data represent mainly Western usage of Kambô and our recruitment did not target those who use Kambô in more ‘ethnographically original’ settings. Moreover, the indigenous traditions in which Kambô, Rapé tobacco and Sananga eye drops are used involve motivations, procedures and subjective benefits sometimes strongly differing from Western interpretations and appropriations.

Moreover, data on experiences and outcomes remain strongly subjective. Specifically, it remains unclear to what extent reported effects are influenced by the settings in which the experiences have been made and the participants’ expectations regarding the substance.

## Conclusion

In conclusion, our survey represents the first study to report quantitative data on the motivation for the use of Kambô, the secretion of *Phyllomedusa bicolor*, and the acute, subacute and long-term effects in Western users. The vast majority of users claimed beneficial effects including changes in consumption patterns towards a more health-orientated behaviour, whereas long-lasting negative effects leading to help-seeking behaviour were reported only by few users. Albeit Kambô use is often associated with the use of nature-derived psychedelics, no acute psychedelic effects were reported. On the other hand, subacute and long-lasting effects were reported, which in some respects resembled afterglow phenomena (Majić et al., 2015) and aspects of subjectively appraised spiritual growth as reported after the use of serotonergic psychedelics.

Questions for future research are whether the effects are mainly due to expectations and the ceremony/setting (psychologically mediated) or if (some) effects also have a direct physiological correlate, which might provide inspiration for the development of new therapeutic approaches.

## Supplemental Material

sj-pdf-1-jop-10.1177_0269881121991554 – Supplemental material for Connected to the spirit of the frog: An Internet-based survey on Kambô, the secretion of the Amazonian Giant Maki Frog (Phyllomedusa bicolor): Motivations for use, settings and subjective experiences Click here for additional data file. Supplemental material, sj-pdf-1-jop-10.1177_0269881121991554 for Connected to the spirit of the frog: An Internet-based survey on Kambô, the secretion of the Amazonian Giant Maki Frog (Phyllomedusa bicolor): Motivations for use, settings and subjective experiences by Tomislav Majić, Meike Sauter, Felix Bermpohl and Timo T Schmidt in Journal of Psychopharmacology

sj-pdf-2-jop-10.1177_0269881121991554 – Supplemental material for Connected to the spirit of the frog: An Internet-based survey on Kambô, the secretion of the Amazonian Giant Maki Frog (Phyllomedusa bicolor): Motivations for use, settings and subjective experiences Click here for additional data file. Supplemental material, sj-pdf-2-jop-10.1177_0269881121991554 for Connected to the spirit of the frog: An Internet-based survey on Kambô, the secretion of the Amazonian Giant Maki Frog (Phyllomedusa bicolor): Motivations for use, settings and subjective experiences by Tomislav Majić, Meike Sauter, Felix Bermpohl and Timo T Schmidt in Journal of Psychopharmacology
