# Supplementary material for: Connected to the spirit of the frog: An Internet-based survey on Kambô, the secretion of the Amazonian Giant Maki Frog (Phyllomedusa bicolor): Motivations for use, settings and subjective experiences
Source: J Psychopharmacol. 2021 Mar 4;35(4):421–36. doi: 10.1177/0269881121991554 (PMC8058834; doi:10.1177/0269881121991554)
Supplement: sj-pdf-1-jop-10.1177_0269881121991554 – Supplemental material for Connected to the spirit of the frog: An Internet-based survey on Kambô, the secretion of the Amazonian Giant Maki Frog (Phyllomedusa bicolor): Motivations for use, settings and subjective experiences [file sj-pdf-1-jop-10.1177_0269881121991554.pdf]

KAMBÔ SURVEY

What's the study about?
This online survey is intended for participants who have received a Kambô treatment at least once, and designed to evaluate their experiences with this practice. In this study, we first inquire about the motivation for receiving Kambô. Furthermore, we are interested in the acute symptoms that occurred during the Kambô sessions as well as the longer-term effects of Kambô treatments. Additionally, there are some questions concerning your personal data such as your age, gender and nationality. You can skip any question you don't want to answer. Since there is only a limited amount of scientific data available on the use of Kambô, we hope to gain a better understanding of the effects and mode of action of Kambô through this study. We kindly ask you to answer the following questions as honestly and as precisely as possible. Questionnaires are anonymous, and no conclusions will be drawn specific to your person. The questionnaire will be used exclusively for this study. You can participate in this study if:
- You have received Kambô at least once in your life
- You are proficient in English
- You are 18 years of age or older

Participating will take you approximately 30 minutes. Thank you very much for taking the time to share your Kambô experiences with us!
Dr. Tomislav Majić Dr. Timo T. Schmidt Meike Sauter

1. **What is your age?**
   … years
2. **What is your gender?**
   Female
   Male
   Other
3. **In which country do you live?**
   Free text
4. **Please indicate your current occupational status**
   Employee
   Freelancer
   Unemployed
   Student (University)
   Student (Secondary School)
   Apprentice
   Retiree
5. **Please indicate your total years of education (incl. secondary school, university, etc.)**
   … years
6. **How old were you when you first received a Kambô treatment?**
   … years
7. **Please indicate how many times you have received Kambô approximately**
   1x
   2-4x
   5-9x
   10-19x
   20-35x
   more than 35x
8. **Please indicate in which setting you have received Kambô and how often (multiple answers possible)**
   At home
   At a friend's place / private space
   With a natural health professional / healer
   At a healing place / temple
   In nature
   In the Amazon rainforest
   At a spiritual festival / healing festival
   Within ceremony with ayahuasca or other psychedelic ritual plants (e.g. peyote, San Pedro, mushrooms, etc.)
   Others:
9. **How long ago was your last Kambô treatment?**
   Less than 1 week ago
   week to 30 days ago
   31 days to 12 months ago
   1-5 years ago
   More than 5 years ago

10. **Which „ritual plants“ or „traditional indigenous medicines“ (such as ayahuasca, peyote, San Pedro, magic mushrooms, etc.) have you ever taken or take occasionally? And, how many times have you taken it in your life?**

Ayahuasca / Yage / Daime
Peyote / San Pedro
Psilocybin / “magic mushrooms”
Iboga
Bufo / 5 MeO-DMT
Rapé / ritual tobacco
Sananga eye drops
Others: …

Never
1x
2-4x
5-10x
11-25x
more than 25x

11. **Which of the following substances have you ever taken or take occasionally? And, how many times have you taken it in your life?**

Alcohol (intoxication)
Opiates (e.g., opium, heroin, morphine, etc.)
Cannabis (e. g., hashish, marihuana)
Cocaine
Amphetamine (“speed”) or methamphetamine (“crystal”)
LSD / LSA
DMT (Dimethyltryptamine) (smoked)
Dissociatives (e.g. ketamine, phencyclidine, PCP, dextromethorphan, DXM)
MDMA (“ecstasy”)
Sedatives (e.g. “valium”) without prescription
Tobacco (smoked)

Others: …
Never
1x
2-4x
5-10x
11-25x
more than 25x

12. **How has the frequency of using these substances changed due to Kambô treatment?**

Never used since then
Less frequent since then
More frequent since then
Unchanged since the Kambô treatment(s)
I don’t have any experience with these substances

13. **Do you feel that Kambô induces an altered state of consciousness?**

1 = Not at all
101 = Profoundly

14. **How often do you engage in spiritual practice (such as meditation, yoga etc.)?**

Daily
Weekly
Monthly
Rarely
Never

15. **Which of the following spiritual practices do you do? And for how long have you practiced?**

Yoga
Meditation
Prayers
Rituals
Mindfulness practice
Breathing exercises
Fasting cure
Being silent
Others: …

16. **How important is/are your spiritual practice(s) to you?**

1 = Not at all
101 = Extraordinary important

17. What were your personal reasons for choosing Kambô treatment? (multiple answers possible)
Substance use disorder / Addiction
Physical weakness and fatigue / chronic fatigue syndrome
Alzheimer disease
Anxiety disorder / Panic disorder
Autoimmune disease in general
Urogenital disorders
Musculoskeletal system issues / back pain (e.g. intervertebral discs)
Negative entity attachment
High blood pressure / low blood pressure
Lyme disease
Chronic inflammations
Chronic pain
Intestinal or other parasites
Depression
Diabetes
Emotional trauma (e.g. due to abuse)
Detoxification after a period of using recreational drugs
Weakened immune system
Emotional imbalance
Hepatitis
HIV / AIDS
Interest in “altered states of consciousness"
Interest in spiritual experience / spiritual growth
Physical detoxification
Any kind of cancer
Gastrointestinal disorders
Manic-depressive disorder
Multiple sclerosis
Negative energies from other people
Curiosity / interest in extreme experiences
Neurodermatitis
Impulsive behaviors
Parkinson disease
Family or relationship problems
Psychosis or schizophrenia
Rheumatological disorders
Improvement of the senses
Thyroid disease
Mental / emotional purification
Sexual dysfunction
Infections of all kinds (viral/bacterial/fungal, e.g. candida)
Other mental or emotional problems
Grief (enduring, pathological)
Improvement of overall performance
Improvement of concentration
Improvement of overall well-being
Desire for general healing
Desire to connect with the spirit of the frog
Being part of a group in which everyone was receiving Kambô
Obsessive-compulsive disorder (OCD)
Others: …

18. What was your most important reason for receiving Kambô for the FIRST time?
Free text

19. Before you FIRST received Kambô, how strongly were you convinced that Kambô would have positive effects on you?
1 = Not at all
101 = Completely convinced

20. In the days after you FIRST received Kambô, how strongly were you convinced that Kambô had actual positive effects on you?
1 = Not at all
101 = Completely convinced

21. To what extent were your expectations for Kambô met after your FIRST treatment?
1 = Not at all
101 = Exceeded all expectations

22. How much have you benefited from your Kambô experience?
1 = Not at all
101 = Extraordinarily

23. **In what ways have you benefitted from Kambô? (multiple answers possible)**
Relief from physical or emotional / mental health problems
Healing of diseases
Spiritual growth
Improvement of physical well-being
Improvement of concentration and mental clarity
Improved emotional health
Others: …
1 = Not at all
101 = Extraordinarily

IN THE FOLLOWING - UNTIL QUESTION 38 - PLEASE FOCUS ON YOUR SUBJECTIVE EXPERIENCE DURING AND AFTER ONE EXEMPLARY KAMBÔ SESSION. IT MIGHT BE A SESSION YOU REMEMBER BEST OR ONE THAT WAS ESPECIALLY MEANINGFUL TO YOU.

24. **How long ago was this session (the one that you remember best or in retrospect was especially meaningful to you)?**
Less than 1 week ago
1 week or more ago
1 month or more ago
1 year or more ago
5 years or more ago
25. **How many "dots" of frog secretion were applied in this Kambô session (the one you remember best or in retrospect was especially meaningful to you)?**
1 2 3 4 5 6 7 8 9 10 11 12 13 14 15 16 17 18 19 20 I don't remember
26. **How did you prepare for this specific Kambô session? (the one you remember best or in retrospect was especially meaningful to you) (multiple answers possible)**
Did not prepare
Defined a certain purpose, intention or goal beforehand
Specific preparation diet
Preparation meeting / conversation with the person who gave the Kambô treatment
Meditation
Yoga
Other spiritual practices
Others: …
27. **Before this exemplary session, did you change your nutritional practices or your intake of recreational drugs? (the session you remember best or in retrospect was especially meaningful to you)**
Yes
No
28. **For which of the following foodstuffs or recreational drugs did you reduce your consumption or dispense with entirely? (multiple answers possible)**
Meat
All kinds of animal products
Fats / oils
Raffinated Sugar
Carbohydrates
Proteins
Alcohol
Tobacco / nicotine
Caffeine
Recreational drugs
Others: …
29. **Did you ingest any ritual plants, other traditional indigenous medicines or recreational drugs during the last 30 days preceding the session?**
Yes
No
I don't remember
30. **Please indicate which plants/medicines/substances you ingested**
Free text
31. **Please indicate the interval between this exemplary session (the one you remember best or in retrospect was especially meaningful to you) and your recent intake of „ritual plants" or other „traditional indigenous medicines".**
0-2 hours
3-12 hours
13-24 hours
2-7 days
8-30 days
32. **What was the setting for this exemplary Kambô session? (the one you remember best or in retrospect was especially meaningful to you) (multiple answers possible)**
At home

At a friend’s place / private space
With a natural health professional / healer
At a healing place / temple
In nature
In the Amazon rainforest
At a spiritual festival / healing festival
Within a ceremony with ayahuasca or other psychedelic ritual plants (e.g. peyote, San Pedro, mushrooms, etc.)
Others: …

33. **Who gave Kambô to you in this exemplary session? (the one you remember best or in retrospect was e especially meaningful to you)**
Indigenous shaman / medicine man
Western shaman / healer / natural health professional
A person without any special shamanic / healing training (layman)
I gave it to myself
Others: …

34. **Who else was there when you received Kambô in this exemplary session? (the one you remember best or in retrospect was especially meaningful to you)**
I was the only client
I was in a group of … other people who also received Kambô
I was alone and applied Kambô to myself

35. **How long did the acute process of this exemplary Kambô session (the one you remember best or in retrospect was especially meaningful to you) last? (without rest after session)**
Less than 15 minutes
15 minutes or more
30 minutes or more
60 minutes or more
90 minutes or more

36. **Which acute symptoms did you experience in this exemplary Kambô session (the one you remember best or in retrospect was especially meaningful to you)? (multiple answers possible)**
Anxiety / panic
Fear of death
Shortness of breath
Stomach pains
Loss of consciousness
Chest pains
Diarrhea
Vomiting
Joy
Whole-body pain
Feeling that the spirit of the frog is with me / present in myself / integrated in my being
Feeling of oneness with everything
Skin changes
Racing heart
Hot flashes / feeling of fever
Cough
Itching
Headache
Back pain
Chills
Swelling of the whole body
Swelling of the trunk
Swelling of arms / legs
Swelling of the face
Swelling of the throat
Dizziness
Sweating
Nausea
Abdominal pain
Increased urination
Desperation
Tremors
Others: …

37. **Looking back on the entirety of your session, please rate the degree to which at any time during that session you experienced the following phenomena. Answer each question according to your feelings, thoughts, and experiences at the time of the session.**
Experience of amazement
Gain of insightful knowledge experienced at an intuitive level

Feelings of tenderness and gentleness
Feelings of peace and tranquility
Sense of reverence
Feeling that you experienced something profoundly sacred and holy
Feelings of joy
Experience of fear
I felt like crying
Feelings of despair
Pressure or weight in my chest or abdomen
Panic
Despair
- none; not at all
- so slight cannot decide
- slight
- moderate
- strong (equivalent in degree to any other strong experience)
- extreme (more than any other time in my life)

38. When considering the LONGER-TERM EFFECTS of this session: What was the duration in which you perceived effects? (the session you remember best or in retrospect was especially meaningful to you)

Up to 3 days
4-7 days
8-30 days
1-6 months
7-12 months
More than 1 year
The effects are noticeable after more than one year and are still there
I don’t remember

IN THE FOLLOWING – UNTIL QUESTION 66 – PLEASE FOCUS ON THE OVERALL EFFECTS THAT KAMBÔ HAS HAD ON YOUR LIFE

39. Which positive longer-term effects has Kambô had for you from your personal viewpoint? (multiple answers possible)

Improvement of physical health
Improvement of mental clarity
Improvement of emotional health
Improvement of general well-being
Improvement of physical issues / physical functions (e.g. digestion, pain, flexibility, decrease of skin disease, etc.)
Improvement of performance
Improvement of spiritual awareness / Increase of spiritual growth
I didn´t notice any positive longer-term effects
- 1 = Not at all
- 101 = Very much

The experience has changed your philosophy of life positively
Your appreciation for life has increased
You have a greater sense of inner authority in your life
Your life has more meaning
You have more enthusiasm for life in general
You are a more authentic person
You have more goodnatured humor / playfulness / lightness
You have more patience / ability to tolerate frustration
You have more optimism
Your self-confidence / self-assurance has increased
Feelings of depression have decreased
Feelings of anxiety have decreased
You have more inner peace (i.e. centeredness, serenity, calmness)
You have a more positive relationship with others
Your negative expression of anger (e.g. ridicule, outward expression of irritability toward others) has decreased.
Your behavior has changed in ways you would consider positive since the experience.
- 1 = none, not at all
- 2 = so slight, cannot decide
- 3 = slight
- 4 = moderate
- 5 = strong
- 6 = extreme

40. Which others positive longer-term effects has Kambô had for you from your personal viewpoint?

Free text

41. **In the days after the Kambô treatment, did any previously non-existing health issues emerge?**
    Yes
    No
    I don't know
42. **Please indicate these new physical problems.**
    Free text
43. **For how long did you perceive these physical problems?**
    Less than 7 days
    7-30 days
    More than 1 month
44. **Were these physical problems so strong and long-lasting that you saw a doctor?**
    Yes
    No
45. **In the days after the Kambô treatment, did any previously existing health issues become more severe, or did they re-emerge?**
    Yes
    No
    I don’t know
46. **Please indicate these well-known physical problems.**
    Free text
47. **For how long did you perceive these well-known physical problems?**
    Less than 7 days
    7-30 days
    More than 1 month
48. **Where these physical problems so strong and long-lasting that you saw a doctor?**
    Yes
    No
49. **In the days after the Kambô treatment, did you experience the emergence of any new mental or emotional problems?**
    Yes
    No
    I don’t know
50. **Please indicate these new mental or emotional problems.**
    Free text
51. **For how long did you perceive these new mental or emotional problems?**
    Less than 7 days
    7-30 days
    More than 1 month
52. **Were these mental or emotional problems so strong and long-lasting that you saw a doctor?**
    Yes
    No
53. **In the days after the Kambô treatment, was there a recurrence of any well-known mental or emotional issues?**
    Yes
    No
    I don’t know
54. **Please indicate these new mental or emotional problems.**
    Free text
55. **For how long did you perceive these mental or emotional problems?**
    Less than 7 days
    7-30 days
    More than 1 month
56. **Where these mental or emotional problems so strong and long-lasting that you saw a doctor?**
    Yes
    No
57. **Do you feel that Kambô has ever led to a long-lasting deterioration of your physical health?**
    Yes
    No
    I don’t know
58. **Do you feel that Kambô has ever led to a long-lasting deterioration of your mental or emotional health?**
    Yes
    No
    I don’t know
59. **After your Kambô treatment, did you change your nutritional habits or your intake of medication, alcohol, tobacco, caffeine or other recreational drugs?**
    Yes
    No

60. **Which of the following foodstuffs, medication, alcohol, tobacco, caffeine or other recreational drugs have you been reducing your intake or dispensing with entirely? And, for how long? (multiple answers possible)**
    Meat
    Alcohol
    Tobacco / nicotine
    Medication
    Recreational drugs
    All kinds of animal products
    Fats / oils
    Raffinated Sugar
    Carbohydrates
    Proteins
    Caffeine
    Others: …
        1-7 days
        8-30 days
        more than 30 days but not permanently
        permanent
        no changes
61. **Have you ever regretted the participation in a Kambô treatment afterwards? (Except during the Kambô session)**
    Yes
    No
    I don’t know
62. **How personally meaningful was the experience?**
    No more than routine, everyday experiences
    Similar to meaningful experiences that occur on average once or more a week
    Similar to meaningful experiences that occur on average once a month
    Similar to meaningful experiences that occur on average once a year
    Similar to meaningful experiences that occur on average once every 5 years
    Among the 10 most meaningful experiences of my life
    Among the 5 most meaningful experiences of my life
    The single most meaningful experience of my
63. **Indicate the degree to which the experience was SPIRITUALLY significant to you.**
    Not at all
    Slightly
    Moderately
    Very much
    Among the 5 most spiritually significant experiences of my life
    The single most spiritually significant experience of my life
64. **Do you believe that the experience and your contemplation of that experience have led to change in your current sense of personal well-being or life satisfaction?**
    Increased very much
    Increased moderately
    Increased slightly
    No change
    Decreased slightly
    Decreased moderately
    Decreased very much
65. **To what extent does the following statement apply to you? “My life has been influenced in a lasting and profound way by Kambô”**
    1 = Not at all
    101 = Extraordinarily
66. **What is the most profound effect Kambô has had on your life?**
    Free text

You have made a valuable contribution to the research on Kambô! Warm thanks for taking the time to share your experience with us. We value your contribution highly. Lead investigators
Dr. Tomislav Majić   Dr. Timo T. Schmidt   Meike Sauter

Questions or Comments? Contact us!
