# Supplementary material for: Connected to the spirit of the frog: An Internet-based survey on Kambô, the secretion of the Amazonian Giant Maki Frog (Phyllomedusa bicolor): Motivations for use, settings and subjective experiences
Source: J Psychopharmacol. 2021 Mar 4;35(4):421–36. doi: 10.1177/0269881121991554 (PMC8058834; doi:10.1177/0269881121991554)
Supplement: sj-pdf-2-jop-10.1177_0269881121991554 – Supplemental material for Connected to the spirit of the frog: An Internet-based survey on Kambô, the secretion of the Amazonian Giant Maki Frog (Phyllomedusa bicolor): Motivations for use, settings and subjective experiences [file sj-pdf-2-jop-10.1177_0269881121991554.pdf]

	Ayahuasca	Peyote	Psilocybin	Iboga	5MeODMT	Rapé	Sananga
never	124 (32.12%)	187 (48.45%)	88 (22.80%)	258 (66.84%)	233 (60.36%)	85 (22.02%)	148 (38.34%)
1x	31 (8.03%)	51 (13.21%)	32 (8.29%)	21(5.44%)	26 (6.74%)	38 (9.84%)	50 (12.95%)
2-4x	73 (18.91%)	57 (14.77%)	71 (18.39%)	8 (2.07%)	33 (8.55%)	63 (16.32%)	52 (13.47%)
5-10x	51 (13.21%)	21 (5.44%)	70 (18.13%)	2 (0.52%)	8 (2.07%)	41 (10.62%)	35 (9.07%)
11-25x	35 (9.07%)	6 (1.55%)	49 (12.69%)	0 (0.00%)	3 (0.78%)	25 (6.48%)	24 (6.22%)
>25x	49 (12.69%)	9 (2.33%)	51 (13.21%)	2 (0.59%)	2 (0.52%)	112 (29.02%)	30 (7.77%)

	Alcohol	Opiates	Cannabis	Cocaine	(Meth)amphetamine	LSD/LSA
never	30 (7.77%)	238 (61.66%)	17 (4.40%)	118 (30.57%)	161 (41.71%)	108 (27.98%)
1x	10 (2.59%)	29 (7.51%)	14 (3.63%)	31 (8.03%)	34 (8.81%)	37 (9.59%)
2-4x	33 (8.55%)	47 (12.18%)	36 (9.33%)	56 (14.51%)	38 (9.84%)	63 (16.32%)
5-10x	27 (6.99%)	14 (3.63%)	27 (6.99%)	44 (11.40%)	32 (8.29%)	55 (14.25%)
11-25x	27 (6.99%)	8 (2.07%)	33 (8.55%)	37 (9.59%)	30 (7.77%)	56 (14.51%)
>25x	257 (66.58%)	26 (6.74%)	258 (66.84%)	75 (19.43%)	75 (19.43%)	55 (14.25%)

	DMT	Dissociatives	MDMA	Sedatives	Tobacco	Others
never	201 (52.07%)	202 (52.33%)	92 (23.83%)	229 (59.33%)	49 (12.69%)	64 (16.58%)
1x	50 (12.95%)	39 (10.10%)	28 (7.25%)	29 (7.51%)	14 (3.63%)	5 (1.30%)
2-4x	50 (12.95%)	43 (11.14%)	42 (10.88%)	35 (9.07%)	34 (8.81%)	8 (2.07%)
5-10x	30 (7.77%)	21 (5.44%)	60 (15.54%)	21 (5.44%)	14 (3.63%)	6 (1.55%)
11-25x	17 (4.40%)	8 (2.07%)	55 (14.25%)	9 (2.33%)	11 (2.85%)	3 (0.78%)
>25x	13 (3.37%)	42 (10.88%)	97 (25.13%)	33 (8.55%)	255 (66.06%)	12 (3.11%)

Supplementary Table S2: Use of ritual plants and substances during lifetime

By using the response option “others”, participants additionally reported the use of “salvia divinorium” (n=4) and “phenethylamine” (n=5), as most common responses.
